# A novel lncRNA LNC_000052 leads to the dysfunction of osteoporotic BMSCs via the miR-96-5p–PIK3R1 axis

**DOI:** 10.1038/s41419-020-03006-7

**Published:** 2020-09-23

**Authors:** Mingyang Li, Rong Cong, Liyu Yang, Lei Yang, Yiqi Zhang, Qin Fu

**Affiliations:** 1 Department of Orthopedics, Shengjing Hospital of China Medical University, Shenyang, China; 2 Department of Obstetrics and Gynecology, Shengjing Hospital of China Medical University, Shenyang, China

**Keywords:** Apoptosis, Long non-coding RNAs, Mesenchymal stem cells, Stem-cell differentiation

## Abstract

Bone marrow-derived mesenchymal stem cells (BMSCs) in postmenopausal osteoporosis models exhibit loss of viability and multipotency. Identification of the differentially expressed RNAs in osteoporotic BMSCs could reveal the mechanisms underlying BMSC dysfunction under physiological conditions, which might improve stem cell therapy and tissue regeneration. In this study, we performed high-throughput RNA sequencing and showed that the novel long non-coding RNA (lncRNA) LNC_000052 and its co-expressed mRNA PIK3R1 were upregulated in osteoporotic BMSCs. Knockdown of LNC_000052 could promote BMSC proliferation, migration, osteogenesis, and inhibit apoptosis via the PI3K/Akt signaling pathway. We found that both LNC_000052 and PIK3R1 shared a miRNA target, miR-96-5p, which was downregulated in osteoporotic BMSCs. Their binding sites were confirmed by dual-luciferase assays. Downregulation of miR-96-5p could restrain the effects of LNC_000052 knockdown while upregulation of miR-96-5p together with LNC_000052 knockdown could improve the therapeutic effects of BMSCs. In summary, the LNC_000052–miR-96-5p–PIK3R1 axis led to dysfunction of osteoporotic BMSCs and might be a novel therapeutic target for stem cell therapy and tissue regeneration.

## Introduction

Bone marrow-derived mesenchymal stem cells (BMSCs) are common multipotent stem cells with self-renewal ability and are regarded as an ideal type of seed cells for regenerative cell therapy^1^. They are easy to harvest from bone marrow and exhibit low immunogenicity due to the negligible expression of immune antigens on their surface as well as their inhibitory effects on major immune cells^2,3^. Moreover, their homing ability, together with directed differentiation and secretion in injured sites, makes them become an attractive cell source in tissue regeneration^4^. Owing to these unique properties, BMSCs are widely employed in the treatment of various diseases, such as acute pulmonary injury and myocardial injury^5,6^.

However, due to the limited amounts of cells that can be collected from one donor and the unavoidable differentiation during in vitro cell culture, a continuous supply of standardized clinical-grade stem cells is urgently required for clinical translation^7^. Some groups attempted to regulate the gene expression of BMSCs to improve their functions. Notably, the mechanism of competing endogenous RNAs (ceRNAs), in which long non-coding RNAs (lncRNAs) act as microRNA (miRNA) sponges to regulate target gene expression, has become a hot topic in the search for novel practical targets^8^. Shang et al.^9^ investigated differentially expressed lncRNAs in BMSCs with or without glucocorticoid treatment by lncRNA microarray analysis. Tang et al.^10^ and Sun et al.^11^ studied the osteogenesis-associated lncRNAs by treating BMSCs with osteogenic reagents.

Nevertheless, all previous studies used inducers to treat BMSCs in vitro to search for differentially expressed lncRNAs, which may be insufficient to simulate the actual changes in BMSCs in vivo. During studying the pathogenesis of postmenopausal osteoporosis, our team found the viability and differentiation properties of BMSCs collected from ovariectomy (OVX)-induced osteoporotic rats were remarkably reduced compared with sham-operated (SHAM) rats. Accordingly, we hypothesized that the changes in gene expression during postmenopausal osteoporosis may be of paramount importance for the biological behavior of BMSCs. Therefore, we explored the transcriptional profiles of OVX BMSCs and SHAM BMSCs by high-throughput RNA sequencing (RNA-seq), and identified that the novel lncRNA, LNC_000052, and its co-expressed mRNA phosphoinositide-3-kinase regulatory subunit 1 (PIK3R1), a negative regulatory subunit of PI3K^12^ as well as an inhibitor of the Akt signaling pathway^13^, were upregulated in OVX BMSCs compared with SHAM BMSCs. Then we conducted a series of experiments to characterize the interaction between LNC_000052 and PIK3R1 and their roles in BMSCs.

## Results

### The novel lncRNA LNC_000052 and its co-expressed mRNA PIK3R1 were upregulated in osteoporotic BMSCs

**Fig. 1:**
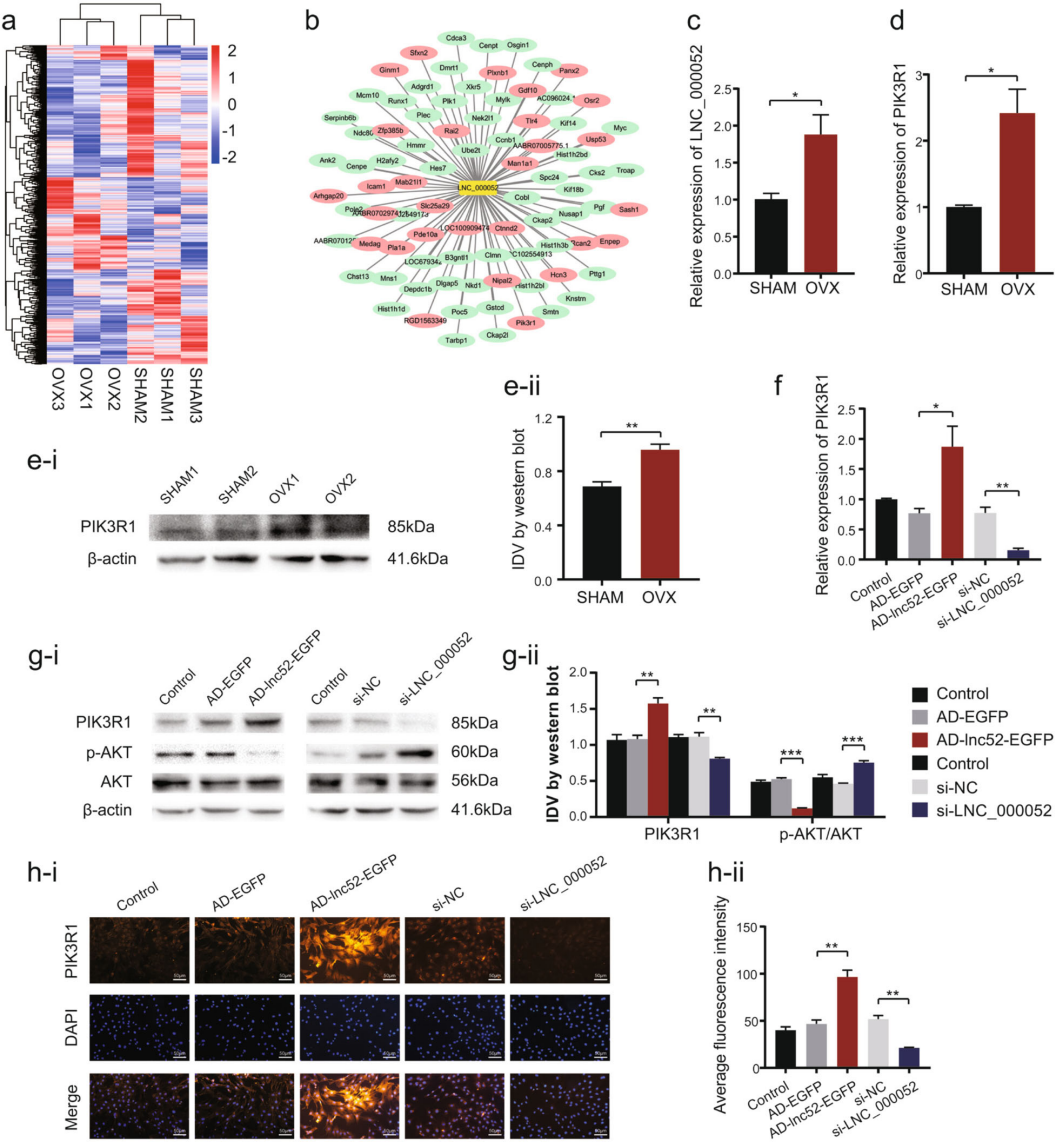
The novel lncRNA LNC_000052 and its co-expressed mRNA PIK3R1 were upregulated in osteoporotic BMSCs. **a** The hierarchical clustering heatmap of lncRNAs that differently expressed in OVX groups and SHAM groups. **b** The LNC_000052-centered co-expression network (*P* < 0.05, |fold change | > 1.5, |Pearson | > 0.96). **c** Relative expression of LNC_000052 in OVX groups and SHAM groups. **d** Relative mRNA expression of PIK3R1 in OVX groups and SHAM groups. **e** Relative protein expression of PIK3R1 in OVX groups and SHAM groups. **f** Relative mRNA expression of PIK3R1 after LNC_000052 regulation. **g** Relative protein expression of PIK3R1 and Akt after LNC_000052 regulation. **h** Immunofluorescence of PIK3R1 after LNC_000052 regulation (image magnification: ×200). Data are presented as the mean ± SEM (*n* = 3 per group). *P < 0.05, **P < 0.01, ***P < 0.0001 vs. NC group.

The RNA-seq analysis provided an overview of the lncRNAs that were differentially expressed in OVX BMSCs compared with SHAM BMSCs. In total, 2541 lncRNAs were markedly differentially expressed in osteoporotic BMSCs, i.e., 849 lncRNAs were upregulated in osteoporotic BMSCs while 1692 were downregulated (Fig. 1a). We aimed to identify some hyper-activated targets that could be inhibited to improve the function of BMSCs for stem cell therapy. Therefore, a novel lncRNA, LNC_000052, and its co-expressed mRNA, PIK3R1, caught our attention (Fig. 1b). We first validated the upregulation of LNC_000052 in OVX BMSCs by qRT-PCR (Fig. 1c). Then we confirmed PIK3R1 was upregulated in OVX BMSCs at both mRNA and protein level (Fig. 1d, e). To prove PIK3R1 was co-expressed with LNC_000052, BMSCs were transfected with an LNC_000052 silencing plasmid (si-LNC_000052) and LNC_000052 overexpression adenovirus (AD-lnc52-EGFP). qRT-PCR, western blot, and immunofluorescence analyses confirmed that the changes in PIK3R1 levels were consistent with those of LNC_000052 (Fig. 1f–h). And the downstream signaling p-Akt/Akt showed an opposite trend against PIK3R1 (Fig. 1g).

### Effects of LNC_000052 on BMSC proliferation, migration, apoptosis, cell cycle, and differentiation

**Fig. 2:**
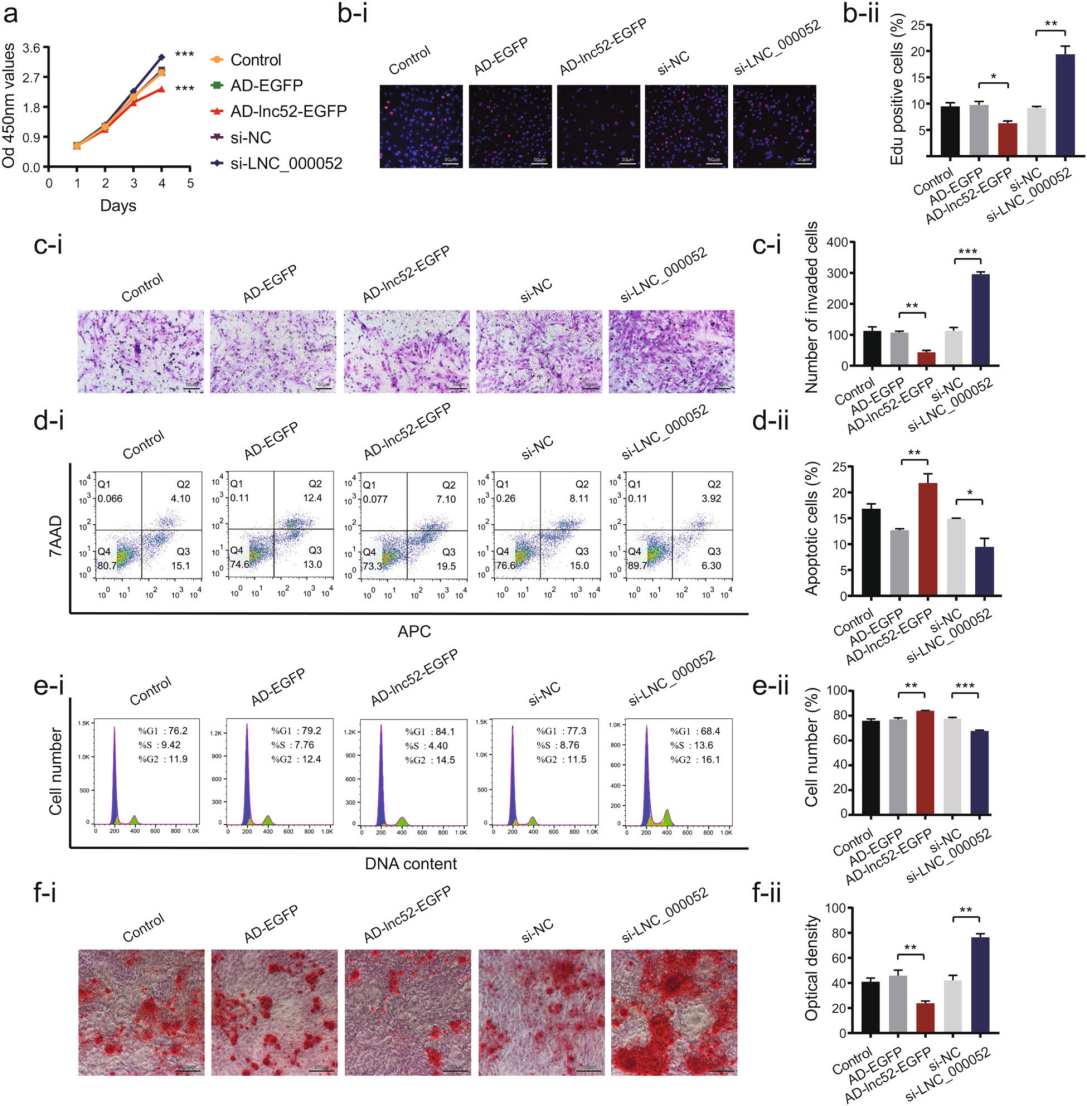
Effects of LNC_000052 on BMSC proliferation, migration, apoptosis, cell cycle, and differentiation. **a** CCK-8 proliferation assays after LNC_000052 regulation. **b** EdU proliferation assays after LNC_000052 regulation. **c** Transwell assays after LNC_000052 regulation. **d** Flow cytometry analysis of cell apoptosis after LNC_000052 regulation. **e** Flow cytometry analysis of cell cycle after LNC_000052 regulation. **f** Alizarin-red staining of LNC_000052-regulated BMSCs after culturing in an osteogenic medium for 14 days (image magnification: ×200). Data are presented as the mean ± SEM (*n* = 3 per group). *P < 0.05, **P < 0.01, ***P < 0.0001 vs. NC group.

Cell Counting Kit-8 (CCK8) and EdU assays demonstrated the proliferation of LNC_000052 overexpressing BMSCs were prominently decreased (Fig. 2a, b). Transwell assays showed that after AD-lnc52-EGFP transfection, the migration ability was significantly decreased (Fig. 2c). Moreover, significantly increased percentages of apoptotic cells and cells in the G0–G1 phase in LNC_000052 overexpressing BMSCs were detected by flow cytometry (Fig. 2d, e). To analyze BMSC multipotency, we took osteogenic differentiation as a reference and found calcium deposition was decreased upon LNC_000052 overexpression (Fig. 2f).

### MiR-96-5p directly bound to PIK3R1 but was sponged by LNC_000052

**Fig. 3:**
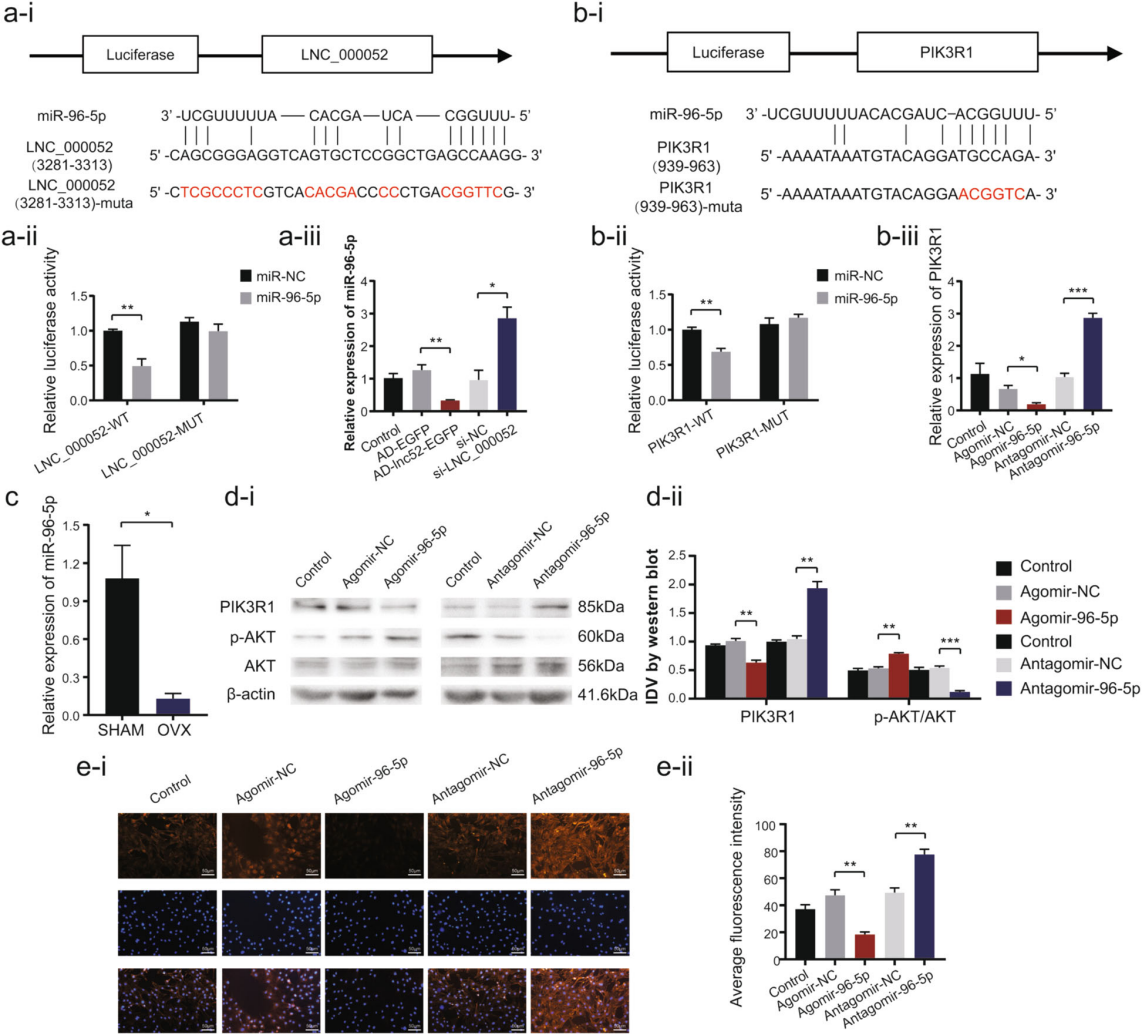
MiR-96-5p directly bound to PIK3R1 but was sponged by LNC_000052. **a** The predicted binding site of LNC_000052 and miR-96-5p was verified by dual-luciferase reporter assays. Relative expression of miR-96-5p was detected after LNC_000052 regulation. **b** The predicted binding site of miR-96-5p and PIK3R1 was verified by dual-luciferase reporter assays. Relative mRNA expression of PIK3R1 was detected after miR-96-5p regulation. **c** Relative expression of miR-96-5p in OVX groups and SHAM groups. **d** Relative protein expression of PIK3R1 and Akt after miR-96-5p regulation. **e** Immunofluorescence of PIK3R1 after miR-96-5p regulation (image magnification: ×200). Data are presented as the mean ± SEM (*n* = 3 per group). *P < 0.05, **P < 0.01, ***P < 0.0001 vs. NC group.

In order to identify miRNAs with binding sites with both LNC_000052 and PIK3R1 that are lowly expressed in osteoporotic BMSCs, we screened the bioinformatics databases TargetScan (http://www.targetscan.org/vert_72/) and Bibiserv (https://bibiserv.cebitec.uni-bielefeld.de/) and identified miR-96-5p as the only eligible miRNA (Fig. 3a–c). Luciferase activity significantly decreased upon co-transfection of miR-96-5p mimics and wild-type LNC_000052 compared with the other three groups. Meanwhile, the co-transfection group with miR-96-5p mimics and wild-type PIK3R1 exhibited prominently decreased luciferase activity compared with the other groups. We further altered LNC_000052 expression and found miR-96-5p expression was negatively correlated with LNC_000052 levels (Fig. 3a-iii). Then we transfected BMSCs with miR-96-5p agomir or antagomir, and found that *PIK3R1* expression was downregulated in agomir-96-5p cells and upregulated in antagomir-96-5p cells, while p-Akt/Akt changed oppositely (Fig. 3b-iii-e). Collectively, miR-96-5p interacts with LNC_000052 and PIK3R1 via these specific binding sites.

### Effects of miR-96-5p on BMSC proliferation, migration, apoptosis, cell cycle, and differentiation

**Fig. 4:**
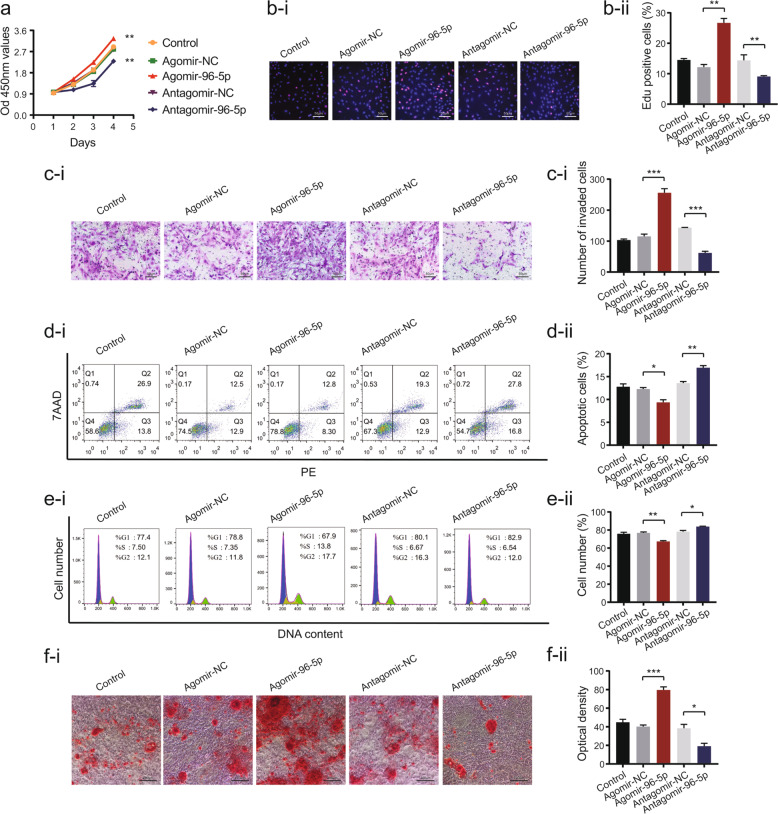
Effects of miR-96-5p on BMSC proliferation, migration, apoptosis, cell cycle, and differentiation. **a** CCK-8 proliferation assays after miR-96-5p regulation. **b** EdU proliferation assays after miR-96-5p regulation. **c** Transwell assays after miR-96-5p regulation. **d** Flow cytometry analysis of cell apoptosis after miR-96-5p regulation. **e** Flow cytometry analysis of cell cycle after miR-96-5p regulation. **f** Alizarin-red staining of miR-96-5p-regulated BMSCs after culturing in an osteogenic medium for 14 days (image magnification: ×200). Data are presented as the mean ± SEM (*n* = 3 per group). *P < 0.05, **P < 0.01, ***P < 0.0001 vs. NC group.

CCK-8 and EdU assays demonstrated that decreases in miR-96-5p expression significantly inhibited cell proliferation (Fig. 4a, b). The migration of BMSCs was inhibited in response to miR-96-5p knockdown (Fig. 4c). Insufficient miR-96-5p also remarkably promoted the apoptosis of BMSCs and increased the percentage of cells in the G0–G1 phase (Fig. 4c, d). Furthermore, antagomir-96-5p significantly delayed BMSC osteogenesis (Fig. 4e). By contrast, miR-96-5p overexpression significantly promoted cell proliferation, migration, osteogenesis, and inhibited apoptosis.

### Knockdown of miR-96-5p restrained the positive effects of LNC_000052 downregulation on BMSCs

**Fig. 5:**
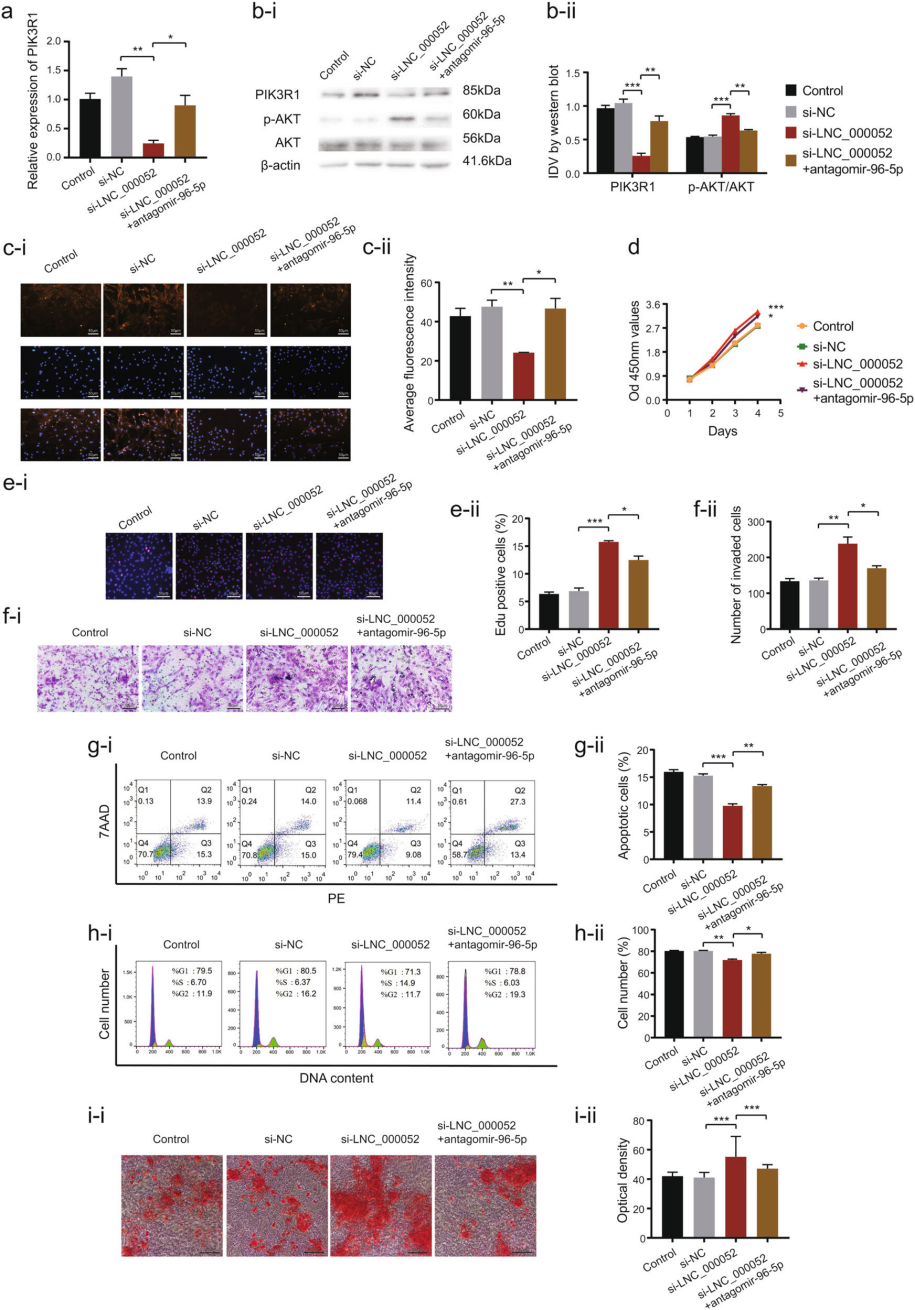
Knockdown of miR-96-5p restrained the positive effects of LNC_000052 downregulation on BMSCs. **a** Relative mRNA expression of PIK3R1 after co-transfection with si-LNC_000052 and antagomiR-96-5p. **b** Relative protein expression of PIK3R1 after co-transfection with si-LNC_000052 and antagomiR-96-5p. **c** Immunofluorescence of PIK3R1 after co-transfection with si-LNC_000052 and antagomiR-96-5p. **d** CCK-8 proliferation assays after co-transfection with si-LNC_000052 and antagomiR-96-5p. **e** EdU proliferation assays after co-transfection with si-LNC_000052 and antagomiR-96-5p. **f** Transwell assays after co-transfection with si-LNC_000052 and antagomiR-96-5p. **g** Flow cytometry analysis of cell apoptosis after co-transfection with si-LNC_000052 and antagomiR-96-5p. **e** Flow cytometry analysis of cell cycle after co-transfection with si-LNC_000052 and antagomiR-96-5p. **f** Alizarin-red staining of si-LNC_000052-antagomiR-96-5p co-transfected BMSCs after culturing in an osteogenic medium for 14 days (image magnification: ×200). Data are presented as the mean ± SEM (*n* = 3 per group). *P < 0.05, **P < 0.01, ***P < 0.0001 vs. NC group.

We co-transfected BMSCs with si-LNC_000052 and antagomir-96-5p, and the cells were divided into the following groups: control, si-NC, LNC_000052(-), and LNC_000052(-)+miR-96-5p(-). qRT-PCR, western blot, and immunofluorescence results showed the expression of PIK3R1 was the lowest in the LNC_000052(-) group, but was recovered in the LNC_000052(-)+miR-96-5p(-) group (Fig. 5a–c). Moreover, in the LNC_000052(-) group, the strongest proliferation and migration abilities, the lowest percentage of apoptotic cells and G0–G1 phase cells, and the most calcium deposition were observed (Fig. 5d–i). By contrast, all effects were weakened in the LNC_000052(-)+miR-96-5p(-) group. Therefore, we conclude miR-96-5p mediates the regulatory effects of LNC_000052 on BMSCs.

### BMSCs co-transfected with si-LNC_000052 and agomir-miR-96-5p had better therapeutic effects

**Fig. 6:**
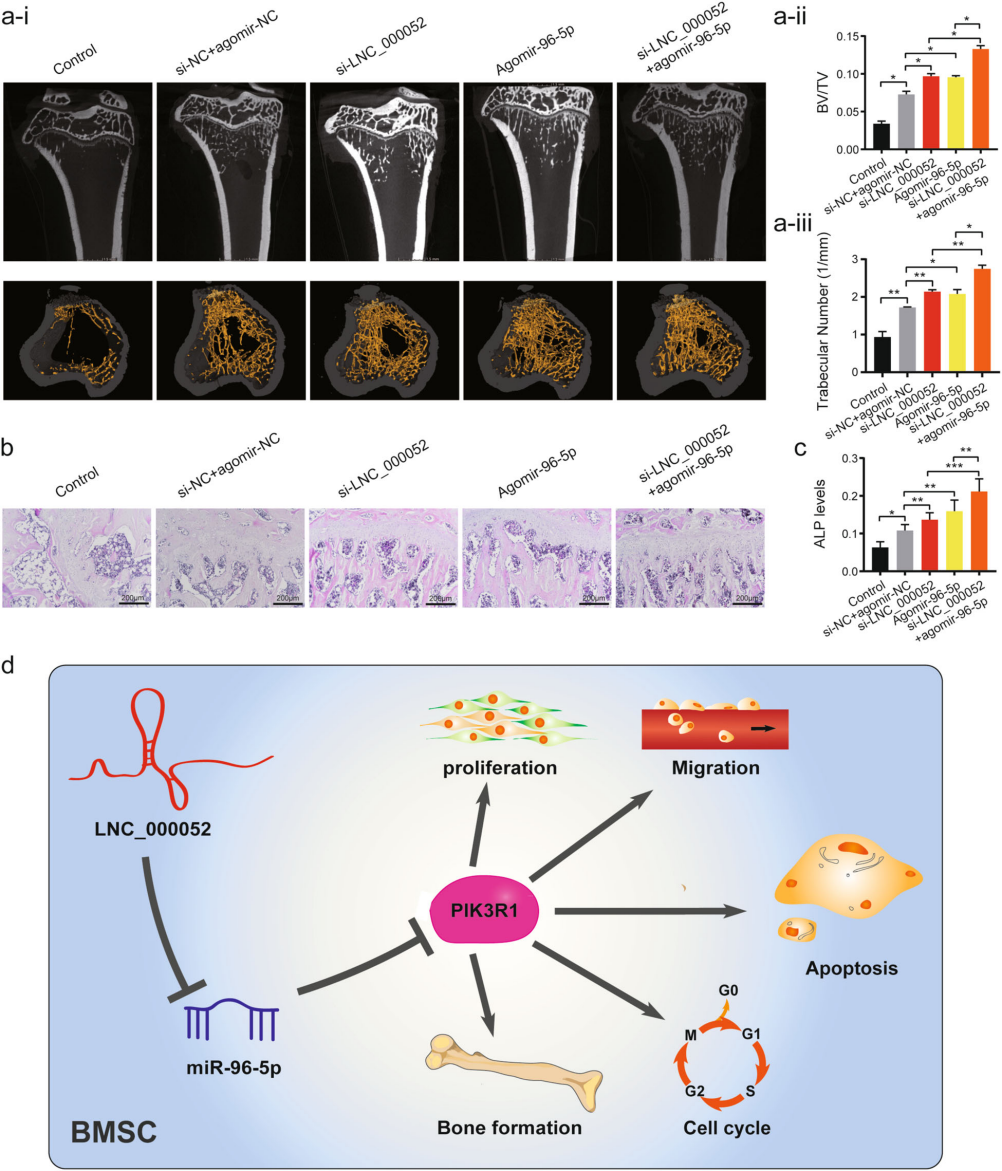
BMSCs co-transfected with si-LNC_000052 and agomir-miR-96-5p had better therapeutic effects. **a** Micro-CT analysis of proximal tibia (the scanning parameters: scanning resolution, 6 μm; voltage, 80 kV; current, 50 μA; exposure time, 600 ms). **b** HE staining of proximal femurs. **c** Serum ALP levels. **d** Schematic of the LNC_000052-miR-96-5p-PIK3R1 axis in the regulation of BMSCs’ biological behaviors. Data are presented as the mean ± SEM (*n* = 3 per group). *P < 0.05, **P < 0.01, ***P < 0.0001 vs. NC group.

To investigate if modulation of LNC_000052 and miR-96-5p levels could improve BMSCs’ therapeutic potential for clinical translation, we established OVX osteoporotic rats and injected the rats with transfected BMSCs. In the treatment groups, thicker cortical bone, increased bone trabecula, less lipid droplets, and better intertrabecular connectivity were observed by micro-CT and hematoxylin–eosin (HE) staining (Fig. 6a, b). Quantitative analysis indicated that the bone volume/tissue volume ratio and the trabecular number were ~25% higher in the LNC_000052(-)+miR-96-5p(+) group compared with the LNC_000052(-) and miR-96-5p(+) groups (Fig. 6a-ii–iii). The changes in serum ALP levels showed both knockdown of LNC_000052 and overexpression of miR-96-5p improved BMSC osteogenesis in vivo, and LNC_000052(-)+miR-96-5p(+) BMSCs exhibited the best therapeutic effects (Fig. 6c).

## Discussion

The dosage of BMSCs for humans in clinical settings is prodigious, and it has been estimated that 10^6^–10^7^ cells/kg are needed per treatment^14^. It is vital to increase the availability and viability of BMSCs for further application. Our group found BMSCs collected from OVX rats exhibited decreased viability and osteogenic ability, which is consistent with a recently published study^15^. On account of these findings, we hypothesized that changes in RNA levels in osteoporotic BMSCs might contribute to the biological behaviors of BMSCs. Thus we conducted RNA-seq analysis.

LNC_000052 was identified as an upregulated novel lncRNA in osteoporotic BMSCs. We performed an LNC_000052-centered co-expression network and found LNC_000052 expression was negatively associated with many important genes affecting cell structure and viability, such as Ccnb1^16^, centromere proteins^17^, and Ckap2^18^, indicating that LNC_000052 overexpression might lead to cell death. Meanwhile, LNC_000052 expression was positively associated with Icam1, a gene which inhibits osteogenesis^19^, and Medag, a gene which promotes adipogenesis^20^, indicating that LNC_000052 might affect BMSC differentiation. In the present study, LNC_000052 overexpression led to reductions in the viability, migration, and differentiation properties of BMSCs, which were consistent with PI3K–AKT pathway dysfunction^21^. Notably, the negative regulatory subunit of PI3K, PIK3R1, was predicted to be a positively co-expressed mRNA of LNC_000052. Therefore, it is of great value to characterize the interaction between LNC_000052 and PIK3R1 and their roles in BMSCs.

Peng^22^ classified the functional mechanisms of lncRNAs in MSCs into the following categories: epigenetic modification, transcriptional expression, posttranscriptional regulation, and others. Considering the changes of PIK3R1 at both mRNA and protein levels, we speculated that LNC_000052 acts as a sponge preventing certain miRNAs from binding to PIK3R1 mRNA. Bioinformatics analysis using TargetScan and Bibiserv suggested that miR-96-5p, which is downregulated in osteoporotic BMSCs, serves as a binding target for both LNC_000052 and PIK3R1. The role of miR-96-5p in BMSCs has not been reported, but it has been identified as an oncogenic miRNA which positively regulates the PI3K/Akt pathway^23,24^. Similarly, our findings suggest that miR-96-5p promotes BMSC proliferation, migration, and osteogenesis and inhibits apoptosis. Furthermore, the binding sites between LNC_000052 and miR-96-5p and between PIK3R1 and miR-96-5p were confirmed by dual-luciferase reporter assays. qRT-PCR showed that miR-96-5p downregulated the expression of PIK3R1 but was downregulated by LNC_000052 overexpression. We conclude PIK3R1 is a target of miR-96-5p and LNC_000052 serves as a ceRNA sponging to miR-96-5p.

Likewise, BMSCs benefited from knockdown of LNC_000052 or overexpression of miR-96-5p. We downregulated LNC_000052 and miR-96-5p at the same time to determine if miR-96-5p mediates the function of LNC_000052. We found that knockdown of miR-96-5p restrained the promotion of viability, migration, and osteogenesis by LNC_000052 downregulation in BMSCs. Moreover, we studied whether the combination of LNC_000052 knockdown and miR-96-5p overexpression could improve the therapeutic effects of BMSCs in vivo. The increased serum ALP levels, bone volumes, and trabecular numbers in the combination group indicated promising improvements in stem cell therapy.

In general, PI3K-Akt is a well-known pathway required for osteogenesis and PIK3R1 usually negatively regulates downstream p-Akt/Akt in most studies^13,25–27^. In Wu’s study^28^, the functions of PIK3R1 (p85α) were examined in BMSCs harvested from p85α^−/−^ mice. In parallel to our findings, it suggested that deletion of p85α increased proliferation, promoted G1–G2/S phase transition, and inhibited apoptosis in BMSCs. Interestingly, deletion of p85α reduced mineralization although the Akt pathway was activated. Wu et al.^28^ attributed this to hyperactivation of the ERK1/2–MAPK signaling pathway, but it has been reported that activation of ERK1/2 signaling could promote BMSC osteogenesis^29^. We noticed that in p85α^−/−^ groups, p110ɑ/β levels were declined. In fact, p85ɑ on the one hand binds to the N-terminus of p110ɑ and inhibits PI3K signaling activity. On the other hand, p85ɑ stabilizes p110ɑ and protects it from degradation^30^. In most cases, p85ɑ levels are much more than p110 levels, and free p85ɑ monomer acts as a negative regulator of p85–p110 heterodimers^31^. Partial p85ɑ knockdown selectively reduces the amount of free p85 levels, and free p85ɑ monomer acts as a negative regulator of p85–p110 heterodimers, and activates PI3K/Akt signaling^32^. By contrast, complete PIK3R1 depletion by a CRISPR/Cas9 strategy destroys the stabilization of p110 and PI3K signaling but the Akt signaling is also activated. This might contribute to the decreases in both PTEN expression and Gab2 phosphorylation^33^. PTEN is a well-known negative regulator of PI3K signaling. Deletion of p85 might in turn weaken PTEN expression^34^ and it has been proved in many tumors that loss of PTEN drives Akt hyperactivation^35^. Moreover, when p85ɑ is partly deleted, Akt activation consequently phosphorylates GSK3β at Ser9, inactivates GSK3β, and destroys the β-catenin degradation complex, thus β-catenin accumulates and activates downstream transcription factors such as Runx2 to increase osteogenesis^36^. When p85ɑ is completely deleted, more phosphorylated GSK3β and accumulated β-catenin are found but the cells present a stem-like phenotype with higher self-renewal ability^37^. The controversy might contribute to the negative feedback loop of GSK3β/PTEN/PI3K/Akt. PTEN deficiency could enhance the phosphorylation of Akt and GSK-3β in response to certain stimuli^38^. The loop might be associated with two highly homologous co-activators, CBP and p300^39–41^, whose interactions with β-catenin are essential for stem cells to either maintain multi-potency and self-renewal or initiate a differentiation program^42^. Thus, we speculate that different stimulus intensities or stimulus methods from upstream signaling might destroy the balance of CBP and p300, and lead to different fate decisions of BMSC. For application in stem cell therapy, the promising findings from experiments with RNA-modified BMSCs might be more valuable and useful.

Some scholars also focused on the RNA modification of BMSCs. However, as mentioned, all previous studies used inducers to treat BMSCs in vitro to search for novel lncRNAs, or referred to well-known lncRNAs studied in other diseases^43^, which might be insufficient to simulate the actual changes in vivo. The OVX rat model has been widely accepted for the study of postmenopausal osteoporosis, which is approved by the US Food and Drug Administration (FDA)^44^. It’s highlight that we performed RNA-seq analysis to screen for differentially expressed RNAs in OVX-induced osteoporotic rats. This study provides dozens of novel pathology-related RNAs and improves our understanding of potential therapeutic strategies for stem cell therapy. Besides the pivotal role of the LNC_000052–miR-96-5p–PIK3R1 axis, we also found notable changes in the expression of some differentiation-related genes, such as Icam1 and Medag, as mentioned above. In view of the above controversy with respect to the multipotency of BMSCs, it might be more beneficial for stem cell therapy to modulate both viability-related and differentiation-related genes simultaneously. The underlying mechanisms of the above targets and some efficient combination therapies deserve further study.

To examine the therapeutic effects of RNA-modified BMSCs, OVX rats were intravenously injected with BMSCs. This method has been widely applied both in the lab and in clinical trails benefiting from BMSCs’ homing capacity to injured tissues^6,45^. However, the migration and homing of BMSCs are affected by various factors, such as chemokines, mechanical stress, microgravity, and the first-pass retention in some tissues^4,46^. The present study suggests that the LNC_000052/miR-96-5p/PIK3R1 axis influences the migration of BMSCs. Moreover, RNA-seq revealed some LNC_000052-related genes functionally involved in BMSC homing, such as PGF^47^, which is worthy of in-depth study.

In summary, we found a novel lncRNA, LNC_000052, which contributes to the dysfunction of osteoporotic BMSCs. LNC_000052 knockdown promoted BMSC proliferation, migration, and osteogenesis and inhibited apoptosis. We further identified the LNC_000052–miR-96-5p–PIK3R1 axis as a critical factor in the regulation of BMSCs’ biological behaviors and discussed its potential for application in stem cell therapy and tissue regeneration based on the successful in vivo experiments (Fig. 6d). Importantly, further screening for differentially expressed RNAs in other pathological BMSCs might shed more light on the understanding of disease pathogenesis and provide more advanced targets and therapeutics for clinical translation.

## Methods

### Osteoporosis model establishment and isolation of BMSCs

Female 8-week-old specific pathogen-free Sprague Dawley (SD) rats were randomly divided into the OVX group (*n* = 20) and the SHAM group (*n* = 20). After acclimatization for 1 week, the rats in the OVX group underwent bilateral OVX, while similar sizes of adipose tissue near the ovaries were resected in the SHAM group. All rats were housed under standard temperature and humidity, with a 12/12-h light/dark cycle and free rodent diet.

After 12 weeks, all rats were euthanized. BMSCs were aseptically harvested from bilateral femurs and tibiae and then incubated using Dulbecco’s modified Eagle medium/nutrient mixture F-12 (DMEM/F12, Hyclone, UT, USA) supplemented with 10% fetal bovine serum (FBS; BioInd, Kibbutz Beit Haemek, Israel) and 1% penicillin–streptomycin solution (BioInd) in a 5% CO_2_ humidified atmosphere at 37 °C. Only third-generation BMSCs were used for RNA-seq. For further verification experiments, the third–fifth-generation BMSCs collected from 3 to 4-week-old female SD rats were used.

### High-throughput sequencing analysis

Third-generation BMSCs collected from three OVX rats and three SHAM rats were frozen in liquid nitrogen and sent to Novogene Bioinformatics Technology Co., Ltd. (Beijing, China) for high-throughput sequencing analysis. RNA-seq was carried out following standard Illumina protocols. Briefly, total RNAs from BMSCs were isolated using TRIzol reagent (Invitrogen, CA, USA) and treated with the Epicentre Ribo-zero™ rRNA Removal Kit (Epicentre, WI, USA) to remove rRNA. Subsequently, sequencing libraries were generated using the rRNA-depleted RNA by the NEBNext^®^ Ultra™ Directional RNA Library Prep Kit for Illumina^®^ (NEB, USA). The products were purified by the AMPure XP system and the library quality was assessed on the Agilent Bioanalyzer 2100 system. After cluster generation using the TruSeq PE Cluster Kit v3-cBot-HS (Illumina, NEB, USA), the libraries were sequenced on an Illumina HiSeq 2500 platform and 125 bp paired-end reads were generated. Data analysis was conducted by the Novogene Gene Regulation Department (Beijing, China).

### RNA extraction and quantitation

Total RNA was extracted from BMSCs using TRIzol (Invitrogen) and reverse transcribed into cDNA using the Primescript™ RT reagent kit (RR047A, TaKaRa, Dalian, China) or the Mir-X miRNA First Strand Synthesis Kit (638315, Takara). The cDNA was used for qRT-PCR using the SYBR Premix Ex Taq kit (RR047A, Takara) with an ABI Prism 7500 Fast Real-Time PCR system (Applied Biosystems, StepOnePlus, USA). GAPDH served as an internal reference for mRNA and lncRNA quantitation, and U6 snRNA was used for miRNA. The relative expression of RNAs was calculated by the 2^−ΔΔCt^ method. All primers were synthesized by Sangon Biotech (Shanghai, China). Primer sequences are listed in Table S1.

### Western blot analysis

BMSCs were lysed using RIPA protein extraction reagent (Beyotime, Beijing, China) supplemented with protease inhibitors and phosphatase inhibitors (Beyotime). The concentrations of extracted proteins were measured by a BCA Protein Assay Kit (Beyotime). The proteins were separated by 10% sodium dodecyl sulfate polyacrylamide gel electrophoresis (SDS–PAGE) and transferred onto polyvinylidene difluoride (PVDF) membranes (Millipore, MA, USA). After blocking with 5% bovine serum albumin (BSA) for 2 h, the membranes were incubated with primary antibodies at 4 °C overnight. The primary antibodies are listed in Table S2. Subsequently, the membranes were incubated at 37 °C with horseradish peroxidase (HRP)-conjugated goat anti-mouse or HRP-conjugated goat anti-rabbit for 2 h. Protein bands were visualized with an Enhanced Chemiluminescence Kit (Beyotime) and analyzed using Quantity One Imaging Software (Bio-Rad, CA, USA).

### Transfection of BMSCs

The si-LNC_000052, rno-miR-96-5p agomir, rno-miR-96-5p antagomir, and their respective negative controls (NCs) were designed and synthesized by GenePharma (Shanghai, China). The sequences are listed in Table S3. BMSCs were transfected with Lipofectamine 3000 reagent (Invitrogen) following the manufacturer’s instructions. The AD-lnc52-EGFP and negative control adenovirus (AD-EGFP) were generated by HanBio Biotechnology (Shanghai, China). The optimal multiplicity of infection was 150. The transfection efficiency was confirmed by qRT-PCR (Fig. S1).

### Immunofluorescence assay

Transfected cells were seeded onto round coverslips in a 12-well plate. The cells were fixed with 4% paraformaldehyde and permeabilized with 0.5% Triton X-100. After blocking with 5% BSA, the cells were incubated with anti-PIK3R1 (Table S2) in a wet box at 4 °C overnight. The cells were incubated with a Cy3-conjugated secondary antibody (Proteintech, Wuhan, China, SA00009-1) and nuclei were stained with DAPI. The stained cells were observed under a fluorescence microscope (Eclipse NI, Nikon, Japan).

### Dual-luciferase reporter gene assay

The recombinant dual-luciferase reporter vectors, including pmirGLO-LNC_000052-wt, pmirGLO-LNC_000052-mut, pmirGLO-PIK3R1-wt, pmirGLO-PIK3R1-mut, and miR-96-5p mimics, miR-96-5p NC, were synthesized by RiboBio (Guangzhou, China). HEK293T cells were purchased and given authentication from the Shanghai Institute of Cell Biology of the Chinese Academy of Sciences (Shanghai, China). The cells were co-transfected with miRNAs and dual-luciferase vectors using Lipofectamine 3000 reagent (Invitrogen) and incubated for 48 h. Luciferase activity was measured by the Dual-Luciferase Reporter Assay System (Promega, WI, USA).

### Cell proliferation assay

BMSCs were seeded in 96-well plates at a concentration of 1 × 10^5^/ml and transfected after incubation for 24 h. At 0, 24, 48, and 72 h after transfection, 10 μl CCK-8 (Dojindo, Japan) reagent was added to each well. After incubation in the dark for 3 h, the optical density (OD) at 450 nm was measured with a microplate reader (Synergy H1, BioTek, USA). Additionally, cell proliferation was visualized with an EdU Cell Proliferation Assay kit (RiboBio) and a fluorescence microscope (Nikon, Japan).

### Cell migration assay

Transfected cells (5 × 10^4^ cells suspended in 200 µl serum-free medium) were seeded into the upper chambers of 8.0 mm Transwell chambers (Corning, NY, USA). The chambers were placed onto 24-well plates containing 750 μl medium with 10% FBS. After 20 h, the chambers were fixed in 4% paraformaldehyde solution and stained with crystal violet. The cells that had passed through the chambers were photographed using a microscope.

### Apoptosis and cell cycle assays

At 48 h after transfection, cells were suspended at a concentration of 1 × 10^5^/ml and stained with the Annexin V APC/7-AAD or PE/7-AAD Apoptosis Detection Kit (KeyGen Biotech, Nanjing, China). After incubation at room temperature for 15 min, apoptosis was analyzed by flow cytometry (FACSCalibur, BD, USA). For cell cycle analysis, transfected cells were harvested and fixed with 70% ethanol at 4 °C overnight. Then 10 μl RNase A solution and 5 μl PI (Solarbio, Beijing, China) were added to the cells in the dark. After 15 min, the cells were analyzed by flow cytometry.

### BMSC transplantation in OVX rats

OVX rats were established by bilateral OVX. On days 30, 45, 60, and 75 after operation, 2.5 × 10^6^ transfected BMSCs were suspended in 500 μl PBS and injected into the rats via the caudal vein. The rats were divided into five groups: control, NC, LNC_000052(-), miR-96-5p(+), and LNC_000052(-)+miR-96-5p(+). The NC group was injected with BMSCs transfected with agomir-NC and si-NC. The control group was injected with 500 μl PBS without BMSCs. On day 90, the rats were euthanized.

### Osteogenic differentiation and staining assay

Third-generation BMSCs were cultured in high-glucose DMEM containing 10% FBS, 10 nM dexamethasone, 50 mg/ml ascorbic acid, and 10 mM β-glycerophosphate. After 14 days, cells were stained with 1% Alizarin Red (pH = 4.2, Solarbio) following the manufacturer’s instructions.

### Serum ALP and histological analysis

Blood was taken from the abdominal aorta of the rats and centrifuged to collect serum. Serum ALP levels were measured using an alkaline phosphatase kit (Solarbio). The tibiae were fixed with 4% paraformaldehyde and the metaphysis of proximal tibia was measured by micro-CT (Y. Cheetah, YXLON, Germany). Data were analyzed by VG Studiomax 3.0 (Volume Graphics, Germany). The femurs were decalcified for 30 days in 5% EDTA and prepared for HE staining according to the protocols (Solarbio).

### Statistical analysis

All data were analyzed using SPSS 20.0 (IBM, Illinois, USA) and GraphPad Prism 5 (GraphPad, CA, USA). Comparisons between two groups were made by means of the two-tailed Student’s *t*-test, and one-way analysis of variance was used to compare differences among multiple groups. *P* < 0.05 was considered as statistical significance.

## Supplementary information

supplementary figure legend

SUPPLEMENTAL MATERIAL Table S1

SUPPLEMENTAL MATERIAL Table S2

SUPPLEMENTAL MATERIAL Table S3

SUPPLEMENTAL MATERIAL Figure S1

## References

[1] Mao Q (2019). ILK promotes survival and self-renewal of hypoxic MSCs via the activation of lncTCF7-Wnt pathway induced by IL-6/STAT3 signaling. Gene Ther..

[2] Machado CV, Telles PD, Nascimento IL (2013). Immunological characteristics of mesenchymal stem cells. Rev. Bras. Hematol. Hemoter..

[3] Liu D (2018). Circulating apoptotic bodies maintain mesenchymal stem cell homeostasis and ameliorate osteopenia via transferring multiple cellular factors. Cell Res..

[4] Fu, X. et al. Mesenchymal stem cell migration and tissue repair. *Cells* **8**, 784 (2019). 10.3390/cells8080784 PMC6721499 31357692

[5] Zhang L, Li Q, Liu Z, Wang Y, Zhao M (2019). The protective effects of bone mesenchymal stem cells on paraquat-induced acute lung injury via the muc5b and ERK/MAPK signaling pathways. Am. J. Transl. Res..

[6] Chen Q (2020). Bone marrow mesenchymal stem cells alleviate the daunorubicin-induced subacute myocardial injury in rats through inhibiting infiltration of T lymphocytes and antigen-presenting cells. Biomed. Pharmacother..

[7] Ferrari D, Gelati M, Profico DC, Vescovi AL (2018). Human fetal neural stem cells for neurodegenerative disease treatment. Results Probl. Cell Differ..

[8] Tye CE (2015). Could lncRNAs be the missing links in control of mesenchymal stem cell differentiation?. J. Cell. Physiol..

[9] Shang G (2018). Long non-coding RNA TCONS_00041960 enhances osteogenesis and inhibits adipogenesis of rat bone marrow mesenchymal stem cell by targeting miR-204-5p and miR-125a-3p. J. Cell. Physiol..

[10] Tang S (2019). LncRNA-OG promotes the osteogenic differentiation of bone marrow-derived mesenchymal stem cells under the regulation of hnRNPK. Stem Cells.

[11] Sun X (2018). Long non-coding RNA, Bmcob, regulates osteoblastic differentiation of bone marrow mesenchymal stem cells. Biochem. Biophys. Res. Commun..

[12] Wang JX (2020). Silencing of miR-17-5p suppresses cell proliferation and promotes cell apoptosis by directly targeting PIK3R1 in laryngeal squamous cell carcinoma. Cancer Cell Int..

[13] Wu HH (2019). TCF7L2 regulates pancreatic beta-cell function through PI3K/AKT signal pathway. Diabetol. Metab. Syndr..

[14] Galipeau J, Sensebe L (2018). Mesenchymal stromal cells: clinical challenges and therapeutic opportunities. Cell Stem Cell.

[15] Huang T (2020). Inhibition of osteogenic and adipogenic potential in bone marrow-derived mesenchymal stem cells under osteoporosis. Biochem. Biophys. Res. Commun..

[16] Fang L, Du WW, Awan FM, Dong J, Yang BB (2019). The circular RNA circ-Ccnb1 dissociates Ccnb1/Cdk1 complex suppressing cell invasion and tumorigenesis. Cancer Lett..

[17] Hu L (2019). Structural analysis of fungal CENP-H/I/K homologs reveals a conserved assembly mechanism underlying proper chromosome alignment. Nucleic Acids Res..

[18] Yoo BH, Kang DS, Park CH, Kang K, Bae CD (2017). CKAP2 phosphorylation by CDK1/cyclinB1 is crucial for maintaining centrosome integrity. Exp. Mol. Med..

[19] Xu FF (2014). Intercellular adhesion molecule-1 inhibits osteogenic differentiation of mesenchymal stem cells and impairs bio-scaffold-mediated bone regeneration in vivo. Tissue Eng. Part A.

[20] Chang JC (2017). Global molecular changes in a tibial compression induced ACL rupture model of post-traumatic osteoarthritis. J. Orthop. Res..

[21] Ye C (2019). Extracellular IL-37 promotes osteogenic differentiation of human bone marrow mesenchymal stem cells via activation of the PI3K/AKT signaling pathway. Cell Death Dis..

[22] Peng S (2018). An overview of long noncoding RNAs involved in bone regeneration from mesenchymal stem cells. Stem Cells Int..

[23] Wei S (2019). The circRNA circPTPRA suppresses epithelial–mesenchymal transitioning and metastasis of NSCLC cells by sponging miR-96-5p. EBioMedicine.

[24] Zhou HY, Wu CQ, Bi EX (2019). MiR-96-5p inhibition induces cell apoptosis in gastric adenocarcinoma. World J. Gastroenterol..

[25] Baker N, Sohn J, Tuan RS (2015). Promotion of human mesenchymal stem cell osteogenesis by PI3-kinase/Akt signaling, and the influence of caveolin-1/cholesterol homeostasis. Stem Cell Res. Ther..

[26] Takeno, A., Kanazawa, I., Tanaka, K. I., Notsu, M. & Sugimoto, T. Phloretin suppresses bone morphogenetic protein-2-induced osteoblastogenesis and mineralization via inhibition of phosphatidylinositol 3-kinases/Akt pathway. *Int. J. Mol. Sci.* **20**, 2481 (2019). 10.3390/ijms20102481 PMC6566987 31137461

[27] Zhao SJ (2020). Macrophage MSR1 promotes BMSC osteogenic differentiation and M2-like polarization by activating PI3K/AKT/GSK3beta/beta-catenin pathway. Theranostics.

[28] Wu X (2011). p85alpha regulates osteoblast differentiation by cross-talking with the MAPK pathway. J. Biol. Chem..

[29] Abdallah, B. M. & Ali, E. M. Butein promotes lineage commitment of bone marrow-derived stem cells into osteoblasts via modulating ERK1/2 signaling pathways. *Molecules* **25**, 1885 (2020). 10.3390/molecules25081885 PMC7221720 32325749

[30] Yu J (1998). Regulation of the p85/p110 phosphatidylinositol 3’-kinase: stabilization and inhibition of the p110alpha catalytic subunit by the p85 regulatory subunit. Mol. Cell. Biol..

[31] Luo J, Cantley LC (2005). The negative regulation of phosphoinositide 3-kinase signaling by p85 and it’s implication in cancer. Cell Cycle.

[32] Thorpe LM (2017). PI3K-p110alpha mediates the oncogenic activity induced by loss of the novel tumor suppressor PI3K-p85alpha. Proc. Natl Acad. Sci. USA.

[33] Li X (2019). Deregulated Gab2 phosphorylation mediates aberrant AKT and STAT3 signaling upon PIK3R1 loss in ovarian cancer. Nat. Commun..

[34] Taniguchi CM (2010). The phosphoinositide 3-kinase regulatory subunit p85alpha can exert tumor suppressor properties through negative regulation of growth factor signaling. Cancer Res..

[35] Haddadi N (2018). PTEN/PTENP1: ‘Regulating the regulator of RTK-dependent PI3K/Akt signalling’, new targets for cancer therapy. Mol. Cancer.

[36] Huang L (2018). A bone-targeting delivery system carrying osteogenic phytomolecule icaritin prevents osteoporosis in mice. Biomaterials.

[37] Lin Y (2015). PIK3R1 negatively regulates the epithelial-mesenchymal transition and stem-like phenotype of renal cancer cells through the AKT/GSK3beta/CTNNB1 signaling pathway. Sci. Rep..

[38] Jang HD, Noh JY, Shin JH, Lin JJ, Lee SY (2013). PTEN regulation by the Akt/GSK-3beta axis during RANKL signaling. Bone.

[39] Ganner A (2019). CBP-1/p300 acetyltransferase regulates SKN-1/Nrf cellular levels, nuclear localization, and activity in *C. elegans*. Exp. Gerontol..

[40] Zhang JS (2011). Mutant K-Ras increases GSK-3beta gene expression via an ETS-p300 transcriptional complex in pancreatic cancer. Oncogene.

[41] He K (2014). Cancer cells acquire a drug resistant, highly tumorigenic, cancer stem-like phenotype through modulation of the PI3K/Akt/beta-catenin/CBP pathway. Int. J. Cancer.

[42] Ring A, Kim YM, Kahn M (2014). Wnt/catenin signaling in adult stem cell physiology and disease. Stem Cell Rev. Rep..

[43] Hu K (2019). Long noncoding RNA ZBED3-AS1 induces the differentiation of mesenchymal stem cells and enhances bone regeneration by repressing IL-1beta via Wnt/beta-catenin signaling pathway. J. Cell. Physiol..

[44] Johnston BD, Ward WE (2015). The ovariectomized rat as a model for studying alveolar bone loss in postmenopausal women. Biomed. Res. Int..

[45] Cai X, Wang L, Wang X, Hou F (2020). miR-124a enhances therapeutic effects of bone marrow stromal cells transplant on diabetic nephropathy-related epithelial-to-mesenchymal transition and fibrosis. J. Cell. Biochem..

[46] De Becker A, Riet IV (2016). Homing and migration of mesenchymal stromal cells: How to improve the efficacy of cell therapy?. World J. Stem Cells.

[47] Park SJ, Kim KJ, Kim WU, Cho CS (2014). Interaction of mesenchymal stem cells with fibroblast-like synoviocytes via cadherin-11 promotes angiogenesis by enhanced secretion of placental growth factor. J. Immunol..

