# Supplementary material for: A novel lncRNA LNC_000052 leads to the dysfunction of osteoporotic BMSCs via the miR-96-5p–PIK3R1 axis
Source: Cell Death Dis. 2020 Sep 23;11(9):795. doi: 10.1038/s41419-020-03006-7 (PMC7511361; doi:10.1038/s41419-020-03006-7)
Supplement: Supplementary file 1 — supplementary figure legend [file 41419_2020_3006_MOESM1_ESM.doc]

**Supplementary figure legend**

**Supplementary Fig.S1** *The transfection efficiency confirmed by qRT-PCR.* **a** Relative expression of LNC_000052 in LNC_000052 knockdown or overexpression groups. **b** Relative expression of miR-96-5p in miR-96-5p knockdown or overexpression groups. **c-d** Relative expression of LNC_000052 and miR-96-5p in co-transfection experiments. **e-g** Relative expression of LNC_000052, miR-96-5p and PIK3R1 in *in-vivo* experiments.

**Supplementary Table S1** Primer sequences for qRT-PCR

**Supplementary Table S2** Primary antibodies used for the detection of protein expression

**Supplementary Table S3** The siRNA, agomir and antagomir sequences
