# Supplementary material for: A novel lncRNA LNC_000052 leads to the dysfunction of osteoporotic BMSCs via the miR-96-5p–PIK3R1 axis
Source: Cell Death Dis. 2020 Sep 23;11(9):795. doi: 10.1038/s41419-020-03006-7 (PMC7511361; doi:10.1038/s41419-020-03006-7)
Supplement: Supplementary file 2 — SUPPLEMENTAL MATERIAL Table S1 [file 41419_2020_3006_MOESM2_ESM.doc]

**Table S1** Primer sequences for qRT-PCR

| **Name** | **Sequence** |
| --- | --- |
| LNC_000052 | F: TTAGAGGCCACAGGCTCACTTACC  R: CCACCTCTCCAGCACCTTGACTC |
| miR-96-5p | F: cgcTTTGGCACTAGCACATTTTTGCT |
| PIK3R1 | F: CCTGGTGCAGCACAATGACTCC  R: GGTGGCTTGAAGGTGAGGAACTG |
| GAPDH | F: GCACCGTCAAGGCTGAGAAC R: TGGTGAAGACGCCAGTGGA |
| U6 | F: CGGGTTTGTTTTGCATTTCT R: AGTCCCAGCATGAACAGCTT |
