# Supplementary material for: A novel lncRNA LNC_000052 leads to the dysfunction of osteoporotic BMSCs via the miR-96-5p–PIK3R1 axis
Source: Cell Death Dis. 2020 Sep 23;11(9):795. doi: 10.1038/s41419-020-03006-7 (PMC7511361; doi:10.1038/s41419-020-03006-7)
Supplement: Supplementary file 3 — SUPPLEMENTAL MATERIAL Table S2 [file 41419_2020_3006_MOESM3_ESM.doc]

**Table S2** Primary antibodies used for the detection of protein expression

| Name | Manufacturer | Dilution ratio:  (*Western blot; Immunofluorescence*) |
| --- | --- | --- |
| PIK3R1 | Proteintech, Wuhan, China  (60225-1-Ig) | 1:5000; 1:500 |
| AKT | Proteintech, Wuhan, China  (60203-2-Ig) | 1:4000 |
| pAKT (S473) | Proteintech, Wuhan, China  (66444-1-Ig) | 1:5000 |
| β-actin | Abcam, Cambridge, UK  (ab8226) | 1:1000 |
