# Supplementary material for: A novel lncRNA LNC_000052 leads to the dysfunction of osteoporotic BMSCs via the miR-96-5p–PIK3R1 axis
Source: Cell Death Dis. 2020 Sep 23;11(9):795. doi: 10.1038/s41419-020-03006-7 (PMC7511361; doi:10.1038/s41419-020-03006-7)
Supplement: Supplementary file 4 — SUPPLEMENTAL MATERIAL Table S3 [file 41419_2020_3006_MOESM4_ESM.doc]

**Table S3** The siRNA, agomir and antagomir sequences

| **Name** | **Sequence** |
| --- | --- |
| | **Si-LNC_000052** | | --- | | Sense: 5′-GCUCGGUACUUCUUGACUATT-3′  Antisense: 5’-UAGUCAAGAAGUACCGAGCTT-3’ |
| **Si-NC** | Sense: 5′-UUCUCCGAACGUGUCACGUTT-3′  Antisense: 5’-ACGUGACACGUUCGGAGAATT-3’ |
| **Agomir-NC** | 5’-UUCUCCGAACGUGUCACGUTT-3’ Antisense: 5’-ACGUGACACGUUCGGAGAATT-3’ |
| **rno-miR-96-5p agomir** | Sense: 5’-UUUGGCACUAGCACAUUUUUGCU-3’  Antisense: 5’-CAAAAAUGUGCUAGUGCCAAAUU-3’ |
| **Agomir-NC** | Sense: 5’-UUCUCCGAACGUGUCACGUTT-3’  Antisense: 5’- ACGUGACACGUUCGGAGAATT-3’ |
| **rno-miR-96-5p antagomir** | 5’-AGCAAAAAUGUGCUAUGUGCCAAA-3’ |
| **inhibitor-NC** | 5’-UUGUACUACACAAAAGUACUG-3’ |
