# Supplementary figures and images for: A novel lncRNA LNC_000052 leads to the dysfunction of osteoporotic BMSCs via the miR-96-5p–PIK3R1 axis
Source: Cell Death Dis. 2020 Sep 23;11(9):795. doi: 10.1038/s41419-020-03006-7 (PMC7511361; doi:10.1038/s41419-020-03006-7)

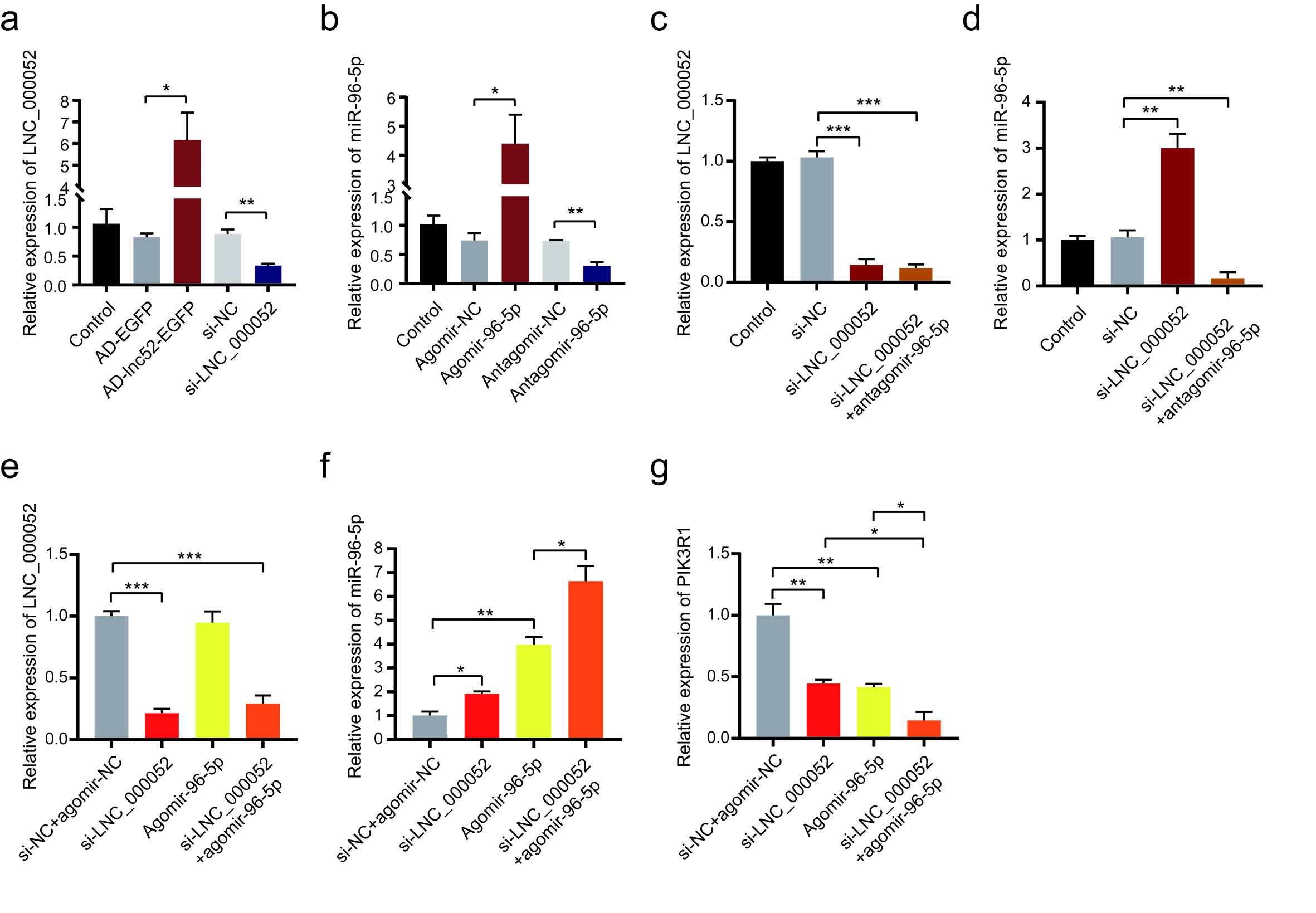

Supplement: Supplementary file 5 — SUPPLEMENTAL MATERIAL Figure S1 [file 41419_2020_3006_MOESM5_ESM.jpg]
